# Vitamin D status and supplementation before and after Bariatric Surgery: Recommendations based on a systematic review and meta-analysis

**DOI:** 10.1007/s11154-023-09831-3

**Published:** 2023-09-04

**Authors:** Andrea Giustina, Luigi di Filippo, Antonio Facciorusso, Robert A. Adler, Neil Binkley, Jens Bollerslev, Roger Bouillon, Felipe F. Casanueva, Giulia Martina Cavestro, Marlene Chakhtoura, Caterina Conte, Lorenzo M. Donini, Peter R. Ebeling, Angelo Fassio, Stefano Frara, Claudia Gagnon, Giovanni Latella, Claudio Marcocci, Jeffrey I. Mechanick, Salvatore Minisola, René Rizzoli, Ferruccio Santini, Joseph L. Shaker, Christopher Sempos, Fabio Massimo Ulivieri, Jyrki K. Virtanen, Nicola Napoli, Anne L. Schafer, John P. Bilezikian

**Affiliations:** 1 Institute of Endocrine and Metabolic Sciences, Università Vita-Salute San Raffaele, IRCCS Ospedale San Raffaele, Via Olgettina 60, 20132 Milan, Italy; 2 Department of Medical and Surgical Sciences, University of Foggia, Foggia, Italy; 3 Richmond Veterans Affairs Medical Center and Virginia Commonwealth University, Richmond, VA USA; 4 University of Wisconsin-Madison, Madison, USA; 5 Faculty of Medicine, University of Oslo, Oslo, Norway; 6 Laboratory of Clinical and Experimental Endocrinology, Department of Chronic Diseases, Metabolism and Ageing, 3000 KU Leuven, Belgium; 7 Molecular Endocrinology Group, Instituto de Investigacion Sanitaria (IDIS), Complejo Hospitalario Universitario de Santiago (CHUS/SERGAS). CIBER de Fisiopatologia de La Obesidad y Nutricion (CIBERobn), Instituto de Salud Carlos III, Madrid, Spain; 8 Gastroenterology and Gastrointestinal Endoscopy Unit, Vita-Salute San Raffaele University, IRCCS San Raffaele Scientific Institute, Milan, Italy; 9 Calcium Metabolism and Osteoporosis Program, American University of Beirut-Medical Center, Beirut, Lebanon; 10 Department of Human Sciences and Promotion of the Quality of Life, San Raffaele Roma Open University, Via Di Val Cannuta 247, 00166 Rome, Italy; 11 Experimental Medicine Department, Sapienza University, Rome, Italy; 12 Department of Medicine, School of Clinical Sciences, Monash University, Clayton, Australia; 13 Rheumatology Unit, University of Verona, Verona, Italy; 14 Department of Medicine, Université Laval, Quebec City, Canada; 15 Gastroenterology, Hepatology and Nutrition Division, Department of Life, Health and Environmental Sciences, University of L’Aquila, L’Aquila, Italy; 16 Department of Clinical and Experimental Medicine, University of Pisa, Pisa, Italy; 17 Kravis Center for Clinical Cardiovascular Health at Mount Sinai Heart, Icahn School of Medicine at Mount Sinai, New York, NY 10029 USA; 18 Sapienza Rome University, Rome, Italy; 19 Service of Bone Diseases, Geneva University Hospitals and Faculty of Medicine, Geneva, Switzerland; 20 Obesity and Lipodystrophy Center, University Hospital of Pisa, Pisa, Italy; 21 Department of Medicine, Medical College of Wisconsin, Milwaukee, WI USA; 22 Vitamin D Standardization Program, Havre de Grace, MD USA; 23 Institute of Public Health and Clinical Nutrition, University of Eastern Finland, Kuopio, Finland; 24 Department of Medicine and Surgery, Research Unit of Endocrinology and Diabetes, Università Campus Bio-Medico di Roma, Via Alvaro del Portillo, 21 - 00128 Roma, Italy; 25 Fondazione Policlinico Universitario Campus Bio-Medico, Via Alvaro del Portillo, 200 - 00128 Roma, Italy; 26 University of California, San Francisco and the San Francisco Veterans Affairs Health Care System, San Francisco, USA; 27 Department of Medicine, Endocrinology Division, Vagelos College of Physicians and Surgeons Columbia University, New York, NY USA

**Keywords:** Bariatric surgery, Obesity, Vitamin D insufficiency, Vitamin D assay, Vitamin D supplementation

## Abstract

Bariatric surgery is associated with a postoperative reduction of 25(OH) vitamin D levels (25(OH)D) and with skeletal complications. Currently, guidelines for 25(OH)D assessment and vitamin D supplementation in bariatric patients, pre- and post-surgery, are still lacking. The aim of this work is to analyse systematically the published experience on 25(OH)D status and vitamin D supplementation, pre- and post-surgery, and to propose, on this basis, recommendations for management. Preoperatively, 18 studies including 2,869 patients were evaluated. Prevalence of vitamin D insufficiency as defined by 25(OH)D < 30 ng/mL (75 nmol/L) was 85%, whereas when defined by 25(OH)D < 20 ng/mL (50 nmol/L) was 57%. The median preoperative 25(OH)D level was 19.75 ng/mL. After surgery, 39 studies including 5,296 patients were analysed and among those undergoing either malabsorptive or restrictive procedures, a lower rate of vitamin D insufficiency and higher 25(OH)D levels postoperatively were observed in patients treated with high-dose oral vitamin D supplementation, defined as ≥ 2,000 IU/daily (mostly D3-formulation), compared with low-doses (< 2,000 IU/daily). Our recommendations based on this systematic review and meta-analysis should help clinical practice in the assessment and management of vitamin D status before and after bariatric surgery. Assessment of vitamin D should be performed pre- and postoperatively in all patients undergoing bariatric surgery. Regardless of the type of procedure, high-dose supplementation is recommended in patients after bariatric surgery.

**Supplementary Information:**

The online version contains supplementary material available at 10.1007/s11154-023-09831-3.

## Introduction

To date, guidelines on vitamin D status assessment and supplementation are mainly focused on the effect of vitamin D on bone e.g., nutritional rickets, osteoporosis, osteomalacia, and parathyroid disorders, and on kidney diseases [1]. A series of International Consensus Conferences, “Controversies in Vitamin D”, have been held annually since 2017 focusing on these areas. The most recent Conferences in this series, held in Stresa (2021) and Florence Italy (2022), aimed to critically investigate potential extra-skeletal effects of vitamin D [2–9]. The latest Conference highlighted, for example, the need to address clinical settings at high-risk of hypovitaminosis D and/or with an impairment of vitamin D metabolism and absorption [9–11]. In particular, the bidirectional relationship between vitamin D and obesity, as well as the impact of bariatric surgery on vitamin D metabolism were considered. In fact, Stresa Consensus participants chose the topic of vitamin D in bariatric surgery as the first one to address and to make specific therapeutic recommendations. Each member of the investigative team was assigned to one of three topics: A) “Vitamin D in obesity”; B) “Assessment of vitamin D status pre- and post-bariatric surgery”; C) “Vitamin D supplementation after bariatric surgery”.

This paper summarizes the work of these three panels. Based on this systematic review and the resulting discussion of the results during the Florence consensus conference, we propose recommendations for management of vitamin D status before and after bariatric surgery.

## Background

### Vitamin D and obesity

Adipose tissue, a direct target of vitamin D, influences its synthesis, distribution, metabolic and endocrine functions [12]. The vitamin D receptor (VDR), present in pre-adipocytes and adipocytes, in both visceral and subcutaneous adipose tissue [13], serves as the mechanistic interface of these properties. Recent *in vitro* studies on mouse adipocytes showed that vitamin 1-25(OH)_2_D_3_, the active form of vitamin D, increases basal and stimulated lipolysis and decreases lipogenesis [14]. The result of these two actions is a catabolic reduction in adipocyte number and size by decreasing lipid and triglycerides accumulation. Moreover, vitamin D affects insulin action and glucose metabolism, by increasing glucose transport in adipocytes through enhanced GLUT4 translocation [15]. Additionally, vitamin D reduces inflammation in adipose tissue. In both preadipocytes and adipocytes, 1-25(OH)_2_D_3_ suppresses expression of multiple inflammatory cytokines, including IL-6, IL-1β and IL-8 [16].

Several pathophysiological mechanisms have been proposed to explain the widely recognized association between vitamin D insufficiency and obesity [17–19]. Patients with obesity tend to spend less time in outdoor physical activity and, thus, have limited skin exposure to sunlight. Lower dietary intake of vitamin D, impaired hepatic 25-hydroxylation [20], impaired hydroxylation in adipose tissue, and alterations in vitamin D receptors [17–19] are additional factors. Excess body fat can also serve as a repository of storage forms of fat-soluble vitamin D (e.g., 25-hydroxvitamin D and parent vitamin D) altering the kinetics between that depot and the circulation. Thus, in addition to the afore mentioned mechanisms that help to account for low circulating levels of 25(OH) vitamin D (25(OH)D) in obesity, sequestration in adipose tissue is another key contributing factor [21, 22].

Patients with obesity typically demonstrate low levels of 25(OH)D, which are inversely correlated with body mass index (BMI) and adiposity [21, 22]. This dynamic adversely affects skeletal and muscle health, resulting in a predisposition to the so-called obese osteo-sarcopenic phenotype [10, 23, 24]. The prevalence of vitamin D insufficiency is reported to be 35% higher in individuals with obesity than in normal weight individuals [21]. Moreover, obese patients often require larger amounts of vitamin D supplementation than their normal-weight counterparts. A recent meta-analysis showed that, after administration of equal doses of vitamin D in patients with obesity, 25(OH)D levels were lower by about 15.2 ng/mL (38 nmol/L) compared with eutrophic individuals, with doses ranging from 4,000–6,000 to 40,000–60,000 IU weekly [25]. Daily vitamin D doses of 4,000 IU may be needed to prevent vitamin D insufficiency in obesity [26].

These pathophysiological aspects of vitamin D metabolism in obesity provide context to the challenge of managing patients who undergo bariatric surgery.

### Bariatric surgery

Therapeutic approaches to severe obesity include lifestyle and nutritional interventions, pharmacotherapy, and bariatric surgery. Although bariatric surgery is not a novel treatment [27, 28], the approach has gained in popularity, over the past several decades, due in part to impressive evidence that weight reduction can be sustained, metabolic comorbidities such as diabetes ameliorated or even cured, and survival improved [27]. Current guidelines recommend bariatric surgery as an option in patients with a BMI ≥ 35 kg/m^2^, regardless of presence, absence, or severity of comorbidities that have not responded to non-surgical strategies [28].

Bariatric surgical procedures reduce body weight in different, but specific ways: restrictive procedures, in which the size of the gastric pouch is greatly reduced; malabsorptive procedures, in which malabsorption of nutrients mostly contributes to weight loss; and a combination of these two approaches [28]. Neurohormonal factors additionally contribute to weight loss and metabolic improvement [29].

The most widely used bariatric surgery procedures are the laparoscopic sleeve gastrectomy (SG) and the laparoscopic Roux-en Y gastric bypass (RYGB). In SG, approximately 80% of the body of the stomach is resected, creating a tubular stomach based on its minor curvature. In RYGB, the stomach is transected, creating a gastric pouch of approximately one ounce capacity and a Roux-en-Y gastrojejunostomy, thus diverting ingested nutrients from the body of the stomach, duodenum, and proximal jejunum directly to the jejunum [28]. SG is considered as a restrictive procedure primarily with a reduced negative impact on nutrient absorption [27]. On the other hand, RYGB promotes weight loss by a combination of malabsorptive and restrictive effects [27]. Another procedure is the biliopancreatic diversion with duodenal switch (BPD). This complex operation involves a SG and an anastomosis to bypass absorptive intestinal sites. BPD is performed less frequently nowadays due to the severe nutrient malabsorption and a higher incidence of short- and long-term complications [28]. Other bariatric procedures include the adjustable gastric band (AGB) and the vertical banded gastroplasty [30]. The jejunoileal bypass, characterized by an intestinal bypass in which the proximal jejunum is bypassed into the distal ileum and, thus, results in extreme weight loss by way of profound nutritional malabsorption, has essentially been abandoned due to its profound short- and long-term malnutritional consequences [27]. Of the currently used bariatric surgical procedures, SG is the most performed worldwide, having overtaken RYGB in popularity about a decade ago [31, 32]. One-anastomosis gastric bypass (OAGB, or mini bypass) has recently emerged as an effective alternative procedure [33]. In recent years, less invasive endoscopic procedures (bariatric endoscopy) have also been developed and introduced in the treatment of obesity [34].

## Aim of the recommendations

Patients with obesity undergoing bariatric surgery typically have low preoperative 25(OH)D levels. Postoperatively, even lower 25(OH)D levels are often observed irrespective of the type of bariatric procedure, although further reductions are particularly marked in malabsorptive procedures [1]. This worsened vitamin D status is accompanied by reduced intestinal calcium absorption and associated bone loss, the latter being multifactorial but importantly related to calcium malabsorption [1, 35].

Currently, there are available expert opinions but no specific guidelines and no international consensus on strategies and goals for vitamin D assessment and supplementation in bariatric patients, pre- and post-surgery.

The aim of this study is to provide evidence-based recommendations on management and achievement of vitamin D sufficiency, before and after bariatric surgery.

## Material and methods

The systematic reviews and recommendations were organized into the following three sections: Section A: Vitamin D in obesity; Section B: Assessment of vitamin D status pre- and post-bariatric surgery; Section C: Vitamin D supplementation after bariatric surgery. Each section was the responsibility of three specific groups of participants. For each group, a coordinating leader permitted smooth operational flow of information: J.P.B. for Section A; A.G. for Section B; A.F. for Section C. After constitution of the groups, each panel member was asked to provide keywords for their section’s literature search. All participants were also asked to provide one or more clinical questions felt to be relevant for their specific group. After the literature search was completed and subjected to initial internal screening in duplicate, each participant received all papers for validation of appropriateness and for exclusion if duplicates or for other reasons. At the end of the screening process, a qualitative assessment of each paper was conducted in duplicate and independently with specific tool, followed by meta-analysis. Meta-analysis and systematic review of the literature was conducted in accordance with the Preferred Reporting Items for Systematic Reviews and Meta-Analyses (PRISMA) checklist [36, 37].

Thresholds for defining vitamin D insufficiency varied among the different studies analysed.

Many studies used the < 30 ng/mL (75 nmol/L) value of 25(OH)D to define vitamin D insufficiency according to the Endocrine Society guidelines [38]. Other studies utilized the < 20 ng/mL (50 nmol/L) threshold, according to the Institute of Medicine (IOM) guidelines [39]. A few studies used both the < 30 and the < 20 thresholds. All studies using the same threshold were analysed together. The characteristics of the studies and the thresholds used are specified in Section 5.

25(OH)D concentrations are reported in ng/mL. To convert ng/mL to nmol/L use the following formula: nmol/L = ng/mL*2.5.

Bariatric restrictive surgical procedures included SG and AGB [27, 28]; bariatric malabsorptive surgical procedures included RYGB and BPD [27, 28]. Mainly malabsorptive, such as BPD, and combined malabsorptive and restrictive procedures, such as RYGB, were pooled together for the analyses.

### Clinical questions and outcomes assessed and PICOs description

The clinical questions were initially proposed by members of each of the three sections. Then, the Consensus group assessed the final formulation of clinical questions focused on the two main topics: 1) assessment of vitamin D status pre- and post-bariatric surgery; 2) supplementation with vitamin D post-bariatric surgery.

For these two major areas, we evaluated the following key clinical questions and calculated pooled rates for the possible relative outcomes extrapolated with the available data and studies (Table 1).

**Table 1:** Key clinical questions for the two topics are provided in this table

**First Topic**	
**Assessment of vitamin D status pre- and post-bariatric surgery**	
*Key clinical questions*	*Outcomes*
1) Should 25(OH)D levels be assessed before bariatric surgery?	1.1 Preoperative assessment of prevalence of 25(OH)D < 30 ng/mL and 25(OH)D < 20 ng/mL 1.2 Preoperative assessment of 25(OH)D levels
2) Should 25(OH)D levels be assessed after bariatric surgery? Do 25(OH)D levels change after bariatric surgery without specific postoperative supplementation?	1.1 Postoperative assessment of prevalence of 25(OH)D < 30 ng/mL and 25(OH)D < 20 ng/mL
3) Is there a difference between restrictive and malabsorptive surgery* in postoperative vitamin D status? ^*^ s*urgical procedures were categorized as malabsorptive (RYGB and BPD) versus (vs) restrictive (AGB and SG)*	1.1 Odds ratios of 25(OH)D < 30 ng/mL and 25(OH)D < 20 ng/mL in restrictive vs malabsorptive surgery at different timepoints (6, 12 and 24 months postoperatively)

**Second Topic**	
**Supplementation with vitamin D post-bariatric surgery**	
*Key clinical questions*	*Outcomes*
1) What dose of vitamin D is necessary for most patients who have undergone bariatric surgery (RYGB, SG, AGB, BPD) to achieve and maintain 25(OH)D levels of ≥ 30 ng/mL? 2) Does the type of bariatric surgery influence the dose of vitamin D supplement required?	1.1 Prevalence of 25(OH)D < 30 ng/mL and 25(OH)D < 20 ng/mL with high-dose vs low-dose supplementation*, among those who underwent malabsorptive procedures AND those who underwent restrictive procedures (at < 6 months and 6–24 months); 1.2 25(OH)D levels with high-dose vs low-dose supplementation, among those who underwent malabsorptive procedures AND those who underwent restrictive procedures (at < 6 months and 6–24 months) * High vitamin D supplementation was defined as a daily supplementation with ≥ *2,000 UI of vitamin D3. Low vitamin D supplementation was defined as a daily supplementation with* < *2,000 UI of vitamin D3*
3) Is there a role for intramuscular (im) vitamin D administration vs oral vitamin D supplementation?	1.1 High- and low-dose im supplementation vs high- and low-dose oral supplementation in malabsorptive AND in restrictive surgery on prevalence of 25(OH)D < 30 ng/mL AND 25(OH)D < 20 ng/mL at different timepoints (< 6 months and 6–24 months) 1.2 High- and low-dose im supplementation vs high- and low-dose oral supplementation in malabsorptive AND in restrictive surgery on 25(OH)D levels at different timepoints (< 6 months and 6–24 months)

The meta-analysis followed PICO methodology [37], including for the first topic comparisons of the pooled data among original studies, observational, interventional, and randomized controlled trials (RCTs), reporting the prevalence of 25(OH)D < 30 ng/mL and 25(OH)D < 20 ng/mL, and 25(OH)D levels in patients undergoing bariatric surgery, pre- and post-surgery without routine postoperative vitamin D supplementation.

The PICO format for our first topic was as follows; (A) Patients: adults with obesity before and after bariatric surgery; (B) Intervention: pre and postoperative prevalence of vitamin D insufficiency using both insufficiency thresholds of < 30 and < 20 ng/mL, respectively, and 25(OH)D absolute levels; (C) Comparator: not applicable; (D) Outcome: pre and postoperative prevalence of 25(OH)D < 30 ng/mL and 25(OH)D < 20 ng/mL, and 25(OH)D levels.

The second topic of our meta-analysis also followed the PICO format [37] and included the indirect comparisons not based on head-to-head studies but on the indirect analyses of pooled estimates of single cohort studies and the pooled data among original studies, observational, interventional and RCTs, reporting the different options of vitamin D supplementation and the prevalence of vitamin D insufficiency using both insufficiency thresholds of < 30 and < 20 ng/mL, respectively, and 25(OH)D levels in patients after bariatric surgery: (A) Patients: adults with obesity after bariatric surgery; (B) Intervention: vitamin D supplementation after bariatric surgery; (C) Comparator: not applicable; (D) Outcome: postoperative prevalence of 25(OH)D < 30 ng/mL and 25(OH)D < 20 ng/mL, and 25(OH)D levels.

### Inclusion and exclusion criteria

The inclusion and exclusion criteria for study selection were based on the two main criteria: 1) measurement of circulating 25(OH)D levels pre- and post-bariatric surgery; 2) Vitamin D supplementation post-bariatric surgery.

The analyses included data from RCTs, case–control, and cohort studies that reported outcomes of interest. The studies were included irrespective of whether they were performed in the inpatient or outpatient setting, country of origin, and follow-up if they provided the appropriate data needed for the analysis. Studies not published in English, small case series (< 10 patients), studies published only as conference abstracts, unpublished works, oral or poster presentations, review articles, and studies using animal models were excluded.

### Search strategy

The relevant medical literature was searched by a medical librarian (F.C.) for studies reporting on the outcomes of interest for the two topics of the meta-analysis. For each Section the search strategy was created using a combination of keywords and standardized index terms (complete list in the supplementary materials). A literature search was conducted on PubMed, EMBASE, Cochrane Library, and Google Scholar, including all studies fulfilling the inclusion criteria published through the end of February 2022. Relevant reviews and meta-analyses in the field were examined for potential additional suitable studies. Two investigators (L.d.F., AG.) independently selected articles of interest based on the aforementioned inclusion and exclusion criteria.

### Data extraction and quality assessment

Data on study participants, intervention-related characteristics, and study-related outcomes from the individual studies were abstracted into a standardized form by two investigators (L.d.F., A.G.), independently. The quality score was assessed by another third author independently (A.F.) based on the Newcastle Ottawa scoring (NOS) system for the observational and interventional studies, and based on the Cochrane tool risk of bias scoring system for RCTs [40, 41]. The overall quality of NOS was based on Selection, Comparability and Outcome criteria, and the Overall Quality identified as Unclear (U), Low (L), Moderate (M), High (H), not available (NA). The overall quality of Cochrane tool risk of bias was based on Selection, Performance, Detection, Attrition, Reporting, Other risk of bias criteria identified as Low (L) or High (H).

GRADE criteria were used to rate the quality of evidence derived from the meta-analysis [42].

Identified articles were reviewed and levels of evidence were assigned by the study methodologist (A.F.). The GRADE criteria for rating quality are summarized here: RCTs are considered to have the highest quality of evidence and can be downrated to moderate, low, or very low quality based on risk of bias in the literature, indirectness, imprecision, inconsistency (or heterogeneity) in the data, or publication bias. On the other hand, observational studies are deemed per se to have low quality of evidence. Starting at the lowest rating of the two pairwise estimates the rating of indirect estimates, when direct comparison was not available, can be further downrated for imprecision or intransitivity (dissimilarity between studies in terms of clinical or methodological characteristics) [42]. The summary statements developed from these articles were assigned a grade of recommendations according to a taxonomy ranging from systematic reviews of RCTs or individual large RCTs supporting a strong recommendation, to expert opinion supporting the lowest grade of recommendation (from A = highest to D = lowest).

### Statistical analyses

Study outcomes were pooled by using a random-effects model based on the DerSimonian and Laird test, and results were expressed as an odds ratio (OR) and a 95% confidence interval (CI) in the case of categorical variables, mean difference and 95% CI in the case of continuous variables. The presence of heterogeneity was calculated using I2 tests with an I2 < 20% interpreted as low-level and an I2 between 20 to 50% interpreted as moderate heterogeneity. Any potential publication bias was verified by using a visual assessment of funnel plots. A funnel plot is a simple scatter plot of the intervention effect estimates from individual studies against some measure of each study’s size or precision. All statistical analyses were conducted using RevMan (version 5.0 for Windows; the Cochrane Collaboration, Oxford, UK), OpenMeta [Analyst] software, and R 3.0.2 (R Foundation for Statistical Computing, Vienna, Austria). For all calculations, a two-tailed p value of less than 0.05 was considered statistically significant.

## Results

### First topic: Assessment of vitamin D status pre- and post-bariatric surgery

**Fig. 1:**
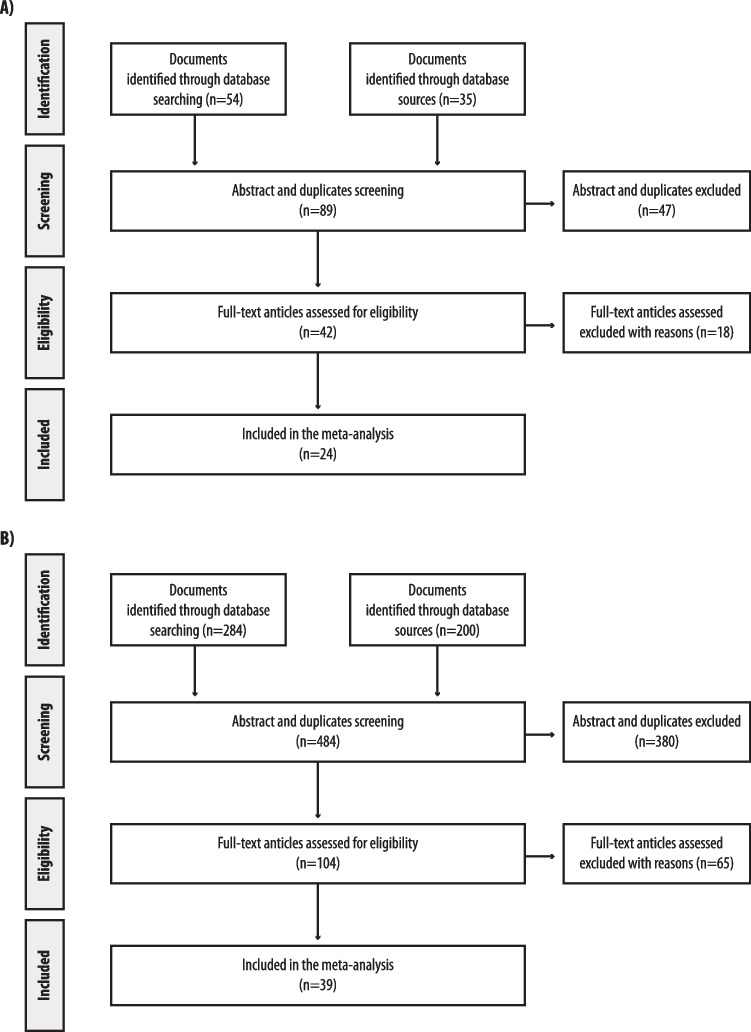
Flow diagram of studies included and excluded in the analyses. **A** Topic 1 “Assessment of vitamin D status pre- and post-bariatric surgery”. **B** Topic 2 “Supplementation with vitamin D post-bariatric surgery”

As shown in Fig. 1A), of 89 papers initially identified, after the exclusion of articles not fulfilling the inclusion criteria, 24 studies [43–66] were included in the meta-analyses. Of 24 included studies, 13 were retrospective [43–45, 48, 49, 53–56, 62–64, 66] and nine were prospective case–control studies [46, 47, 50, 51, 57–61], and two were RCTs [52, 65].

Quality assessment of the studies included in this topic is summarized in Supplementary (Suppl.) Tables 1–3. In the following paragraphs the data analyses related to each key clinical question formulated by the group will be reported.

#### Key clinical question #1: Should 25(OH)D levels be assessed before bariatric surgery?

To answer this question, the prevalence of vitamin D insufficiency using the 25(OH)D < 30 ng/mL and < 20 ng/mL thresholds, and mean circulating 25(OH)D levels before surgery were assessed across the included studies. Of 24 papers initially identified, 18 studies were evaluated [43–60]. Nine were retrospective [43–45, 48, 49, 53–56] and eight were prospective case–control studies [46, 47, 50–52, 57–60], and one was a RCT [52], including a total of 2,869 patients. The studies defining hypovitaminosis D as 25(OH)D levels below 30 ng/mL [44, 45, 47–49, 53] and characterizing the hypovitaminosis D status using both 30 ng/mL and 20 ng/mL thresholds [46, 50–54] were analysed to evaluate the prevalence of 25(OH)D < 30 ng/mL. The studies defining hypovitaminosis D as 25(OH)D levels below 20 ng/mL [55, 56, 58–60] and characterizing the hypovitaminosis D status using both 30 ng/mL and 20 ng/mL thresholds [46, 50–54] were analysed to evaluate the prevalence of 25(OH)D < 20 ng/mL. The studies reporting also the absolute 25(OH)D levels were analysed to evaluate the preoperative 25(OH)D [44, 45, 47, 48, 50, 57–59].

**Fig. 2:**
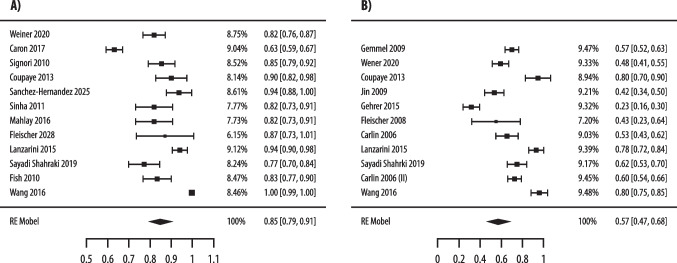
Pooled analysis of preoperative prevalence of vitamin D insufficiency, as defined by 25(OH)D < 30 ng/mL (**A**) and by < 20 ng/mL (**B**), in patients who underwent bariatric surgery

As reported in Fig. 2, the pooled rates of preoperative vitamin D insufficiency in patients who underwent bariatric surgery were 85% (95% CI 79%–91%, I2 = 94.2%), using the < 30 ng/mL threshold (12 studies and 1781 patients) [43–54], and 57% (95% CI 47%–68%, I2 = 95.7%) using the < 20 ng/mL threshold (11 studies and 1765 patients) [43, 46, 50–52, 54–56, 58–60], respectively.

As reported in Suppl. Fig. 1, the mean/median level of preoperative 25(OH)D in patients undergoing bariatric surgery was 19.75 (15.98–23.52) ng/mL (8 studies and 1227 patients, I2 = 88%) [44, 45, 47, 48, 50, 57–59].

Quality of evidence and publication bias of key clinical question #1: most of the studies on which the meta-analysis was based were non-comparative observational studies. Therefore, a recommendation D, with low quality of evidence, due to risk of bias and inconsistency, supported the assessment of 25(OH)D levels in all patients undergoing bariatric surgery. Visual assessment of the funnel plot revealed relative symmetry regarding primary outcome, thus implying low possibility of publication bias for technical success and quality of evidence was downrated only for risk of bias in the literature and inconsistency (Suppl. Fig. 2).

The statement proposed by Consensus group regarding key clinical question #1 was: “25(OH)D levels should be evaluated preoperatively in all patients who undergo bariatric surgery”.

Recommendation D; Low quality evidence.

#### Key clinical question #2: Should 25(OH)D levels be assessed after bariatric surgery? Do 25(OH)D levels change after bariatric surgery without specific postoperative supplementation?

To answer this question, prevalence of vitamin D insufficiency using 25(OH)D < 30 ng/mL and < 20 ng/mL thresholds after surgery was assessed across included studies. Only studies without routine postoperative use of vitamin D were evaluated, and four studies fulfilled inclusion criteria [61–64]. Of four included studies, three were retrospective studies [62–64] and one was a prospective cohort study [61], including a total of 394 patients. One study was analysed to evaluate the prevalence of 25(OH)D < 30 ng/mL [61], and the other three studies were analysed to evaluate the prevalence of 25(OH)D < 20 ng/mL [62–64]. Pooled rates of postoperative vitamin D insufficiency in patients after bariatric surgery (Suppl. Fig. 3) were 63% (95% CI 46%–79%) (1 study and 243 patients, I2 = 92.56%), with < 30 ng/mL threshold [61], and 64% (95% CI 38%–90%) (3 studies and 151 patients, I2 = 92.7%), using < 20 ng/mL threshold [62–64].

Quality of evidence and publication bias for key clinical question #2: since most of included studies were observational, quality of evidence was rated low due to risk of literature bias and inconsistency. The visual assessment of the funnel plot showed relative symmetry regarding primary outcome, thus implying low possibility of publication bias for technical success and quality of evidence was downrated only for risk of literature bias and inconsistency (Suppl. Fig. 4).

The statement proposed by Consensus group regarding key clinical question #2 was: “25(OH)D levels should be routinely evaluated in all patients who have undergone bariatric surgery. Without specific postoperative supplementation, high rates of vitamin D insufficiency are observed”.

Recommendation D; Low quality evidence.

#### Key clinical question #3: Is there a difference between restrictive and malabsorptive surgery in postoperative vitamin D status?

Odds ratios of preoperative and postoperative prevalence of vitamin D insufficiency using the 25(OH)D < 30 ng/mL and < 20 ng/mL thresholds in restrictive procedures versus (vs) procedures with a malabsorptive component (collectively classified as “malabsorptive”) at different timepoints (before surgery and after 6, 12 and 24 months) were evaluated. Of 24 papers initially identified, after exclusion of articles without available specific data, five studies were evaluated [51, 53, 60, 65, 66]. Two were retrospective [53, 66], two prospective case–control studies [51, 60] and one was a RCT [65], including a total of 593 patients. All five studies used routine postoperative vitamin D supplementation ranging from 400 to 3,000 IU/daily. The studies defining hypovitaminosis D as 25(OH)D levels below 30 ng/mL [53, 65, 66] and characterizing hypovitaminosis D status using both 30 ng/mL and 20 ng/mL cut-offs [51] were analysed to evaluate the prevalence of 25(OH)D < 30 ng/mL. The studies defining hypovitaminosis D as 25(OH)D levels below 20 ng/mL [60] and characterizing hypovitaminosis D status using both 30 ng/mL and 20 ng/mL thresholds [51] were analysed to evaluate prevalence of 25(OH)D < 20 ng/mL.

**Fig. 3:**
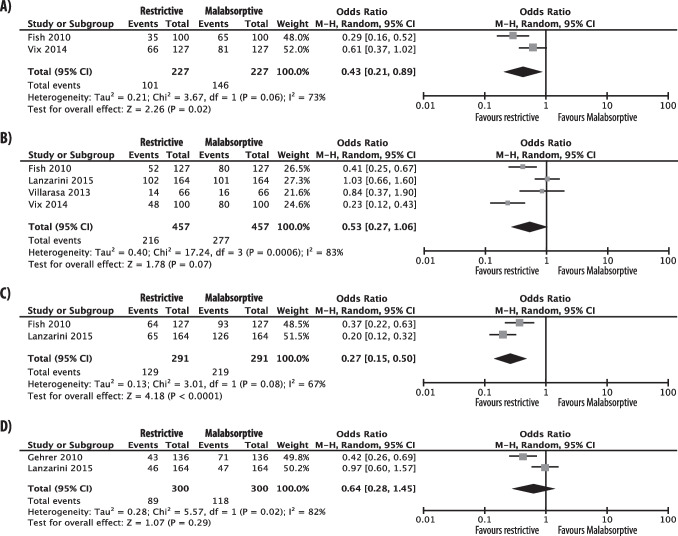
Forest plots comparing restrictive vs malabsorptive surgery in postoperative prevalence of vitamin D insufficiency, as defined by 25(OH)D < 30 ng/mL (**A**-**C**) and by < 20 ng/mL (**D**). Panel **A** analyses at 6-month; panel **B** analyses at 12-month; panel **C** analyses at 24-month; panel **D** analyses at 12-month

Based on four studies [51, 53, 65, 66] (457 patients), significant differences in prevalence of 25(OH)D < 30 ng/mL were found at 6 and 24 months after surgery between the two groups, observing a lower rate in patients who underwent restrictive compared with those who underwent malabsorptive procedures (OR 0.43, 0.21–0.89, p = 0.02, I2 = 73%; OR 0.27, 0.15–0.5, p < 0.0001, I2 = 67%; respectively) (Fig. 3). Odds ratios did not meet statistical significance at the 12-month postoperative timepoint (OR 0.53, 0.27–1.06, p = 0.07, I2 = 83%; respectively) (Fig. 3).

Additionally, analysing data of two studies [51, 60] (300 patients), differences in the prevalence of 25(OH)D < 20 ng/mL between the two groups at 12 months after surgery, were non-statistically significant (OR 0.64, 0.28–1.45, p = 0.29, I2 = 82%) (Fig. 3).

Quality of evidence and publication bias for key clinical question #3: since most of included studies were observational, quality of evidence suggesting different vitamin D outcomes with the two surgical approaches was low, with risk of literature bias and inconsistency. The visual assessment of the funnel plot disclosed relative symmetry regarding prevalence of 25(OH)D < 30 ng/mL outcome, with low possible publication bias for technical success (Suppl. Fig. 5).

The statement proposed by Consensus group regarding key clinical question #3 was: “Patients undergoing malabsorptive bariatric surgery have higher rates of 25(OH)D <30 ng/mL than those undergoing restrictive bariatric surgery”.

Recommendation C; Low quality evidence.

### Supplementation with vitamin D post-bariatric surgery

As shown in Fig. 1 B), of 484 papers initially identified, after the exclusion of articles not fulfilling inclusion criteria, 39 studies [43, 44, 51, 65, 67–101] were included in the meta-analyses. Of them, 16 were retrospective [43, 44, 72, 74, 75, 77, 79, 81, 82, 86, 89, 91, 94, 95, 97, 99], 11 were prospective case–control studies [51, 67–71, 76, 80, 96, 98, 101] and 12 were RCTs [65, 73, 78, 83–85, 87, 88, 90, 92, 93, 100], including a total of 5,296 patients. Quality assessment of the studies included in this topic is summarized in Suppl. Table 4.

#### Key clinical question #1: What dose of vitamin D is necessary for most patients who have undergone bariatric surgery to achieve and maintain 25(OH)D levels of ≥30 ng/mL? and Key clinical question #2: Does the type of bariatric surgery influence the dose of vitamin D supplementation required?

We evaluated the prevalence of vitamin D insufficiency using 25(OH)D < 30 ng/mL and < 20 ng/mL thresholds, as well as 25(OH)D levels in the postoperative setting. Two postoperative timepoints were used: < 6 months and 6–24 months. Treatment doses were categorized as “high” if vitamin D dose was ≥ 2,000 IU/daily, or “low/standard” if < 2,000 IU/daily [1]. Data were also split by intervention type (malabsorptive and restrictive). Studies defining hypovitaminosis D as 25(OH)D levels below 30 ng/mL [44, 69, 72, 74, 76, 79, 83, 84, 97] and characterizing vitamin D status using both 30 ng/mL and 20 ng/mL thresholds [51, 75, 82] were used to evaluate prevalence of 25(OH)D < 30 ng/mL. Studies defining hypovitaminosis D as 25(OH)D levels less than 20 ng/mL [65, 70, 73, 77, 78, 80, 81, 85, 86, 88, 89, 93–95] and characterizing vitamin D status using both 30 ng/mL and 20 ng/mL thresholds [51, 75, 82] were analysed to evaluate prevalence of 25(OH)D < 20 ng/mL. Studies reporting 25(OH)D levels were analysed to estimate postoperative vitamin D status [43, 44, 51, 65, 67–69, 71, 75–87, 89–96, 98–101].

**Fig. 4:**
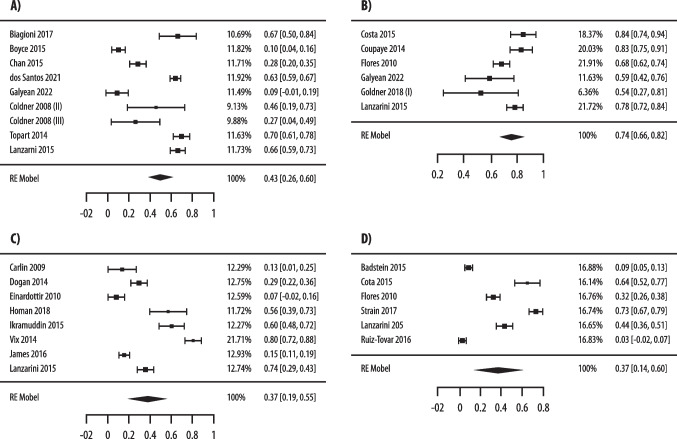
Pooled analysis of postoperative prevalence of vitamin D insufficiency, as defined by 25(OH)D < 30 ng/mL (**A**, **B**) and by < 20 ng/mL (**C**, **D**), in patients at 6–24 months after malabsorptive bariatric surgery treated with high- (**A**, **C**) and low-dose (**B**, **D**) of vitamin D supplementation

A significant difference in prevalence of 25(OH)D < 30 ng/mL (11 studies with 1623 patients) [51, 69, 72, 74–76, 79, 82–84, 97] but not of 25(OH)D < 20 ng/mL (13 studies with 1570 patients) [51, 65, 70, 73, 75, 78, 80, 82, 86, 88, 89, 93, 95] was observed at 6–24 months after malabsorptive surgery. In fact, a significantly different prevalence of 25(OH)D < 30 ng/mL was observed in patients treated with high-dose vs low-dose supplementation (43% vs 74%, p = 0.01) (Fig. 4).

**Fig. 5:**
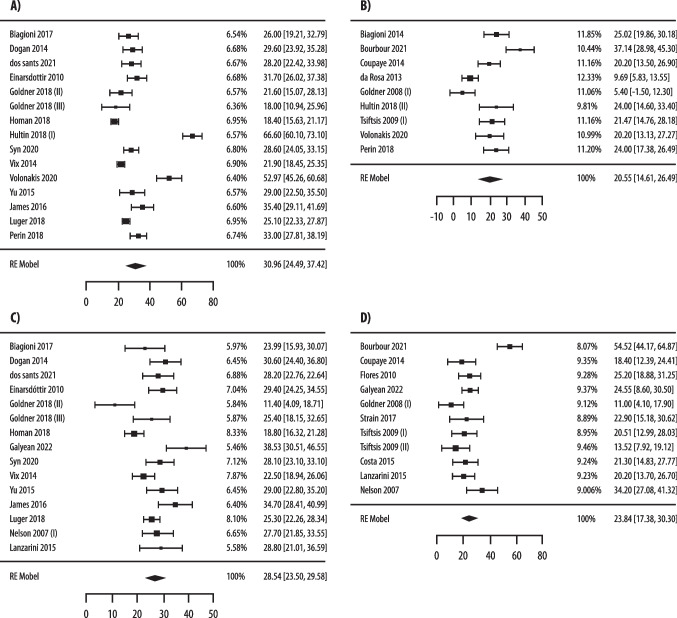
Pooled analysis of postoperative 25(OH)D levels (ng/mL) at < 6 months (**A**, **B**) and 6–24 months (**C**, **D**) after malabsorptive bariatric surgery in patients treated with high- (**A**, **C**) and low-dose (**B**, **D**) of vitamin D supplementation

Based on 19 studies [65, 68, 69, 71, 76–80, 84, 86, 87, 89, 90, 92, 96, 98, 99, 101] (2,490 patients) at < 6 months after malabsorptive surgery higher 25(OH)D levels in patients treated with high- vs low-dose supplementation (30.96 vs 20.55 ng/mL, p = 0.03) were found (Fig. 5). This difference in 25(OH)D levels was not statistically significant in 20 studies [51, 65, 69, 71, 75, 76, 78–80, 82–84, 86, 89–91, 95, 96, 98, 101] (2,432 patients) at 6–24 months (26.54 vs 23.84 ng/mL, p = 0.21) (Fig. 5).

**Fig. 6:**
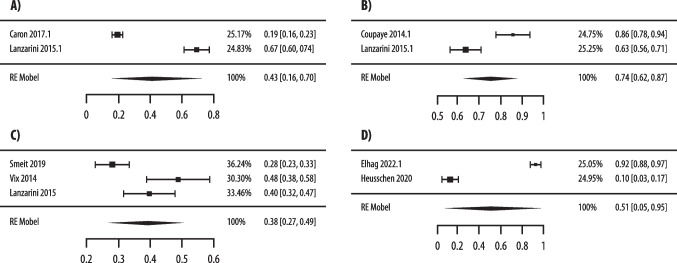
Pooled analysis of postoperative prevalence of vitamin D insufficiency, as defined by 25(OH)D < 30 ng/mL (**A**, **B**) and by < 20 ng/mL (**C**, **D**), in patients at 6–24 months after restrictive bariatric surgery treated with high- (**A**, **C**) and low-dose (**B**, **D**) of vitamin D supplementation

**Fig. 7:**
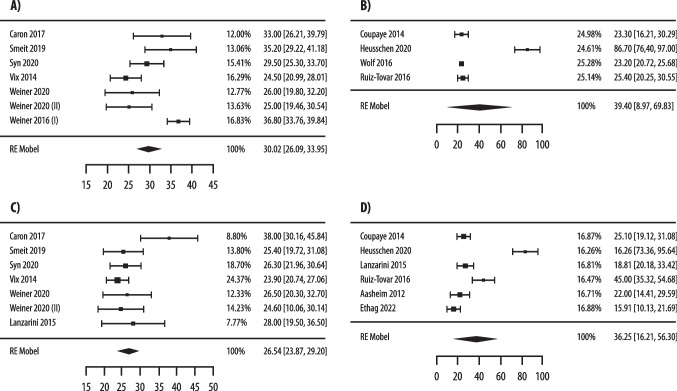
Pooled analysis of postoperative 25(OH)D levels (ng/mL) in patients < 6 months (**A**, **B**) and 6–24 months (**C**, **D**) after restrictive bariatric surgery treated with high- (**A**, **C**) and low-dose (**B**, **D**) of vitamin D supplementation

Significantly different prevalence of 25(OH)D < 30 ng/mL (3 studies, 777 patients) [44, 51, 76] and of 25(OH)D < 20 ng/mL (5 studies, 770 patients) [51, 65, 81, 85, 94] was observed at 6–24 months after restrictive surgery in high vs low-dose groups, with lower rates in patients with high-dose supplementation (43% vs 74%, p = 0.04 and 38% vs 51%, p = 0.05, respectively) (Fig. 6). Conversely, a non-significant difference in 25(OH)D was observed after restrictive surgery between the two groups either at < 6 months (30.02 vs 39.4 ng/mL, p = 0.43) (Fig. 7) [43, 44, 65, 76, 85, 93, 94, 96, 100, 101] (1,801 patients) or at 6–24 months (26.54 vs 36.25 ng/mL, p = 0.55) [43, 44, 51, 65, 67, 76, 81, 85, 93, 94, 96] (2,337 patients) (Suppl. Fig. 6).

In summary, we observed in patients treated with high (≥ 2,000 IU daily) vs low doses (< 2,000 IU daily) of vitamin D: 1) lower rates of 25(OH)D < 30 ng/mL at 6–24 months after either malabsorptive or restrictive surgery; 2) lower rates of 25(OH)D < 20 ng/mL at 6–24 months after restrictive surgery; 3) higher 25(OH)D levels at < 6 months after malabsorptive surgery; 4) among those undergoing restrictive procedures, no differences in 25(OH)D levels at any timepoints. Importantly, even with high-dose supplementation, hypovitaminosis D was common after both forms of bariatric procedures, independently of the biochemical definitions.

Quality of evidence and publication bias for key clinical questions #1 and #2: since most of the included studies were observational and non-comparative retrospective studies, quality of evidence was rated low due to risk of literature bias and inconsistency. Visual assessment of the funnel plot revealed relative symmetry regarding the different outcomes, thus implying, despite the small number of studies, low possibility of publication bias (Suppl. Fig. 6).

The statement proposed by the Consensus group regarding key clinical questions #1 and #2 was: “Postoperative doses of vitamin D supplementation ≥2,000 IU/daily result in lower rates of vitamin D insufficiency (only as defined by 30 ng/mL threshold) compared to doses <2,000 IU/daily, regardless of the type of intervention and timepoints”.

Recommendation D; low quality evidence.

#### Key clinical question #3: Is there a role for intramuscular vitamin D administration versus oral vitamin D supplementation?

Due to lack of data and low number of studies, only descriptive statistics, comparing studies with intramuscular (im) vs oral supplementation could be used for this analysis. Three studies, one RCT, one observational and one prospective interventional [70, 80, 87] specifically evaluated im vitamin D supplementation (259 patients). Levels of 25(OH)D and rates of hypovitaminosis D as defined by 25(OH)D < 20 ng/mL were compared in those with high- and low-dose im supplementation vs high- and low-dose oral supplementation only after malabsorptive surgery at < 6 and 6–24 months.

We observed higher 25(OH)D levels at both < 6 and 6–24 months in those treated with high-dose im vs high-dose oral supplementation (< 6 months: 49.55 vs 30.9 ng/mL; 6–24 months: 29.4 vs 26.5 ng/mL). We observed higher 25(OH)D levels at < 6 months in those treated with low-dose im vs low-dose oral supplementation (29.2 vs 20.55 ng/mL). We also observed lower prevalence of 25(OH)D < 20 ng/mL, at both < 6 and 6–24 months in those treated with high-dose im vs high-dose oral supplementation (< 6 months: 3.7% vs 39%; 6–24 months: 7.5% vs 37%) and lower prevalence of 25(OH)D < 20 ng/mL at < 6 months in those treated with low-dose im vs low-dose oral supplementation (9.1% vs 37%).

The statement proposed by the group regarding clinical question #3 was: “In patients undergoing malabsorptive surgery, use of intramuscular supplementation may be considered an alternative to oral supplementation, as it results in higher 25(OH)D levels and lower rates of vitamin D insufficiency, especially at high-dose”.

Recommendation D; low quality evidence.

Quality of evidence and publication bias for clinical question #3: given lack of data and low number of studies, descriptive statistics were used, and quality of evidence was rated very low, relying mainly on expert opinion.

## Discussion

These are first recommendations focused specifically on clinical management of vitamin D status in obese patients pre- and post-bariatric surgery based on systematic review and meta-analysis of 25(OH)D levels and prevalence of pre- and postoperative hypovitaminosis D in patients with obesity undergoing restrictive and malabsorptive bariatric procedures and supplemented after surgical treatment with different vitamin D doses and routes of administration.

Results obtained through the meta-analyses were evaluated by a panel of international experts who issued several statements on pre-defined clinical questions. Evidence levels of these proposed statements were markedly influenced by inconsistently controlled nature of the studies currently available in the literature and included in the meta-analysis, mostly conducted with only observational and retrospective designs, and evaluating non-randomised patient cohorts. Despite these limitations, this is the first study systematically evaluating the large amount of published data on vitamin D in patients undergoing bariatric surgery, confirming that prevalence of hypovitaminosis D, defined more (25(OH)D < 20 ng/mL, as per IOM guidelines) [39], or less restrictively (< 30 ng/mL, as per Endocrine Society guidelines) [38], is common urgently calling for recommendations on assessment and management of vitamin D status in this population.

### Assessment of vitamin D status pre- and post-bariatric surgery

Statement 1: “*25(OH)D levels should be evaluated preoperatively in all patients who undergo bariatric surgery*”. The statement is based upon meta-analysis data reporting high rate (85%) of preoperative vitamin D insufficiency defined with less restrictive 25(OH)D threshold (< 30 ng/mL) in patients undergoing bariatric surgery. However, even using a more restrictive threshold (< 20 ng/mL) a high rate (57%) of hypovitaminosis D was observed. These results are in line with a prospective follow-up study including data from 164 patients with severe obesity treated with either SG [96] or with RYGB [58], in which adequate 25(OH)D status (> 30 ng/mL) was found in only 5.7% of patients preoperatively. The prevalence of 25(OH)D levels > 20 and < 30 ng/mL, > 10 and < 20 ng/mL and < 10 ng/mL, was 15.1%, 59.1% and 20.1%, respectively [51]. Similar findings were observed in a retrospective study on 211 patients assessed pre-bariatric surgery for nutritional deficiencies, with a rate of hypovitaminosis D up to 80% [54]. The statement highlights widespread hypovitaminosis D in obese patients undergoing bariatric surgery. It is thus recommended to assess preoperative 25(OH)D levels, recognized as the most reliable biochemical marker for defining vitamin D status [3], in all these patients. All candidates for bariatric surgery with vitamin D deficiency should be supplemented according to existing guidelines [4].

Statement 2: “*25(OH)D levels should be routinely assessed in all patients after bariatric surgery. Without postoperative supplementation, high rates of vitamin D insufficiency are observed”.* To date, it is widely accepted that patients require vitamin D supplementation after bariatric procedures although still administered with different doses and types. Thus, we analysed vitamin D status after bariatric surgery in the few available studies in which patients were not vitamin D supplemented in order to properly evaluate postoperative prevalence of hypovitaminosis D. After an extensive literature search, four studies fulfilling these criteria were included in the meta-analyses and showed rates of postoperative vitamin D insufficiency up to 64%. Thus, prevalence of 25(OH)D < 20 ng/mL was higher than that reported in the preoperative setting (57%). These results confirmed that 25(OH)D levels should be routinely evaluated in all patients who have after bariatric surgery and that these patients require specific routine supplementation with vitamin D.

Statement 3: *“Patients undergoing malabsorptive bariatric surgery have higher rates of 25(OH)D* < *30 ng/mL than those undergoing restrictive bariatric surgery”*. Not surprisingly, patients undergoing malabsorptive procedures experience higher risk of post-surgical deficiency of several nutrients as compared to those undergoing restrictive procedures. We systematically compared the impact of procedures with a malabsorptive component with that of purely restrictive procedures on patients’ postoperative prevalence of 25(OH)D < 30 ng/mL and 25(OH)D < 20 ng/mL. In line with what could be expected, but never systematically examined, higher rates of hypovitaminosis D were found in patients after malabsorptive vs restrictive procedures. However, non-significant differences were observed in postoperative prevalence of more stringently defined hypovitaminosis D between malabsorptive and restrictive procedures, with highly significant rates also occurring in patients undergoing restrictive procedures which are generally thought less likely to cause nutritional deficiencies.

### Supplementation of vitamin D post-bariatric surgery

Statement 1: *“Postoperative doses of vitamin D supplementation* ≥ *2,000 IU/daily result in lower rates of vitamin D insufficiency (only as defined by 30 ng/mL threshold) compared to doses* < *2,000 IU/daily, regardless of the type of intervention and timepoints”.*

Although there is general consensus about routinely supplementing with vitamin D post bariatric surgery, in particular after malabsorptive procedures, specific guidance on therapeutic dose regimens and strategies were based on expert opinions or part of general guidelines on bone health in bariatric patients [102]. In our search, we found highly heterogeneous therapeutic approaches ranging from a daily oral intake of 400–800 IU, as also suggested by recommendations for healthy people, to very high doses, currently avoided due to potential side effects [1].

Due to this significant heterogeneity, we categorized different therapeutic options into two main dose regimen categories: low/standard dose of daily oral vitamin D supplementation < 2,000 IU, and high-dose ≥ 2,000 IU. Daily vitamin D supplementation ≥ 2,000 IU was more effective in reducing occurrence of postoperative hypovitaminosis D, even using more restrictive definition, vs daily supplementation with < 2,000 IU after either restrictive or malabsorptive procedures.

Therefore, to prevent potential negative effects of bariatric surgery on skeletal health [34], the group recommended to treat all patients after bariatric procedures with at least 2,000 IU daily vitamin D3 supplementation and to periodically monitor 25(OH)D levels.

The lack of significance of high-dose vitamin D in obtaining 25(OH)D levels > 20 ng/mL in bariatric patients may depend on several factors. In fact, they may include patients with very pronounced lack of vitamin D who may require higher doses or more prolonged treatment vs those used in examined studies. Moreover, due to the lower number of patients meeting this definition as compared to the 30 ng/mL threshold, statistical significance may be more difficult to reach.

Statement 2: *“In patients undergoing malabsorptive surgery, the use of intramuscular supplementation may be considered instead of the oral one, as it results in higher 25(OH)D levels and lower rates of vitamin D insufficiency, especially at a high-dose”.*

As previously discussed, bariatric surgery negatively affects vitamin D status through variable degrees of intestinal malabsorption. The efficacy of oral supplementation is accordingly reduced [31]. To circumvent this therapeutic challenge, other routes of administration may be considered, particularly in those with severe intestinal malabsorption. The parenteral route of vitamin D administration has been shown to be effective and safe in patients with hypovitaminosis D caused by severe intestinal malabsorption [1]. The im route of vitamin D administration, although not always available worldwide, is recommended as first therapeutic choice in several gastrointestinal disorders, including inflammatory bowel diseases, pancreatic insufficiency, short-bowel syndrome, untreated gluten enteropathy, and in need for total parenteral nutrition [1]. For the first time to our knowledge, we have systematically compared the efficacy in increasing 25(OH)D levels and reducing hypovitaminosis D occurrence between high- and low-dose im supplementation vs high- and low-dose oral supplementation in patients undergoing malabsorptive surgery. We observed that the im route was more effective compared with oral administration, resulting in higher 25(OH)D levels and lower rates of vitamin D insufficiency, even when defined with the more restrictive threshold, especially with im high-dose. Therefore, in bariatric surgery, the Consensus expert panel recommends that im vitamin D be considered as a possible preferred route of administration, when available, in patients who undergo malabsorptive procedures [103].

As previously mentioned, most of the studies included in the analyses for vitamin D supplementation after surgery were conducted using vitamin D3 formulations with a single study included evaluating calcifediol use [51]. Also, only three studies [74, 83, 96], two observational and one interventional, specifically evaluated ergocalciferol (vitamin D2) supplementation (854 patients). In these few latter studies, conducted after malabsorptive or restrictive procedures with high-dose ergocalciferol supplementation, postoperative 25(OH)D levels were in the range of the pooled data observed in studies conducted with cholecalciferol supplementation. However, due to the paucity of data it was not possible to perform specific statistical analyses and therefore issue any recommendations on the use of forms of vitamin D other than cholecalciferol.

## Limitations

Studies analysed used two different thresholds for defining vitamin D insufficiency. Therefore, it was not possible to evaluate the potential impact on our data of using lower thresholds that are also used to define hypovitaminosis D [3]. However, the relatively similar results observed in studies using less restrictive thresholds as compared to more stringent ones (as also supported by the few studies in which both thresholds were adopted [46, 50–54, 75, 82]) suggest that profound vitamin D insufficiency occurs throughout the studies in patients undergoing bariatric surgery. On the other hand, the absence of 25(OH)D assay standardization and of assay quality assessment in studies analysed is a relevant limitation in our meta-analysis as in all other similar works in the field of vitamin D [3].

Due to the heterogeneity of the studies and of the data reported, no recommendations could be given concerning the timing of vitamin D assessment, particularly during follow-up. However, most studies assessed 25(OH)D levels within 3 months following the surgical procedure, a reasonable timepoint for decision making on early supplementation with vitamin D. Moreover, due to the lack of data, it was not possible to determine how to best monitor the adequacy of vitamin D supplementation particularly in the postoperative period, and we were not able to identify an exact dose-range for effective vitamin D supplementation as well as a role for forms of vitamin D other than cholecalciferol due to the lack of published evidence. Finally, the literature review included studies enrolling only adult patients and since bariatric surgery is now being done also in adolescents who have not reached peak bone mass, the impact of inadequate vitamin D may be even greater requiring an urgent need to develop guidance on vitamin D supplementation also in such younger patients.

## Conclusions

Our meta-analysis-based consensus should help guide clinical practice in the assessment and management of vitamin D status after bariatric surgery. Pre- and post-bariatric surgery 25(OH)D assessment as well as high-dose cholecalciferol supplementation via either the oral or im route are recommended in patients undergoing bariatric surgery, regardless of the type of procedure. The global implementation of these straightforward recommendations could represent an important first step towards ensuring vitamin D sufficiency for all patients after any bariatric surgery procedure.

### Supplementary Information

Below is the link to the electronic supplementary material.

Supplementary file1 (DOCX 329 KB)

## Data Availability

All authors had full access to all the data in the study and take responsibility for the integrity of the data and the accuracy of the data analysis.

## Abbreviation

25(OH)D: 25(OH) vitamin D
AGB: Adjustable gastric band
BPD: Biliopancreatic diversion with duodenal switch
BMI: Body mass index
CI: Confidence interval
IOM: Institute of Medicine
NOS: Newcastle Ottawa scoring
OR: Odds ratio
OAGB: One-anastomosis gastric bypass
PRISMA: Preferred Reporting Items for Systematic Reviews and Meta-Analyses
RCTs: Randomized controlled trials
RYGB: Roux-en Y gastric bypass
SG: Sleeve gastrectomy
suppl.: Supplementary
vs: Versus
VDR: Vitamin D receptor
