# Correction: Vitamin D status and supplementation before and after Bariatric Surgery: Recommendations based on a systematic review and meta‑analysis

**DOI:** 10.1007/s11154-023-09837-x

**Published:** 2023-09-25

**Authors:** Andrea Giustina, Luigi di Filippo, Antonio Facciorusso, Robert A. Adler, Neil Binkley, Jens Bollerslev, Roger Bouillon, Felipe F. Casanueva, Giulia Martina Cavestro, Marlene Chakhtoura, Caterina Conte, Lorenzo M. Donini, Peter R. Ebeling, Angelo Fassio, Stefano Frara, Claudia Gagnon, Giovanni Latella, Claudio Marcocci, Jeffrey I. Mechanick, Salvatore Minisola, René Rizzoli, Ferruccio Santini, Joseph L. Shaker, Christopher Sempos, Fabio Massimo Ulivieri, Jyrki K. Virtanen, Nicola Napoli, Anne L. Schafer, John P. Bilezikian

**Affiliations:** 1 Institute of Endocrine and Metabolic Sciences, Università Vita-Salute San Raffaele, IRCCS Ospedale San Raffaele, Via Olgettina 60, 20132 Milan, Italy; 2 Department of Medical and Surgical Sciences, University of Foggia, Foggia, Italy; 3 Richmond Veterans Affairs Medical Center and Virginia Commonwealth University, Richmond, VA USA; 4 University of Wisconsin-Madison, Madison, USA; 5 Faculty of Medicine, University of Oslo, Oslo, Norway; 6 Laboratory of Clinical and Experimental Endocrinology, Department of Chronic Diseases, Metabolism and Ageing, 3000 KU Leuven, Belgium; 7 Molecular Endocrinology Group, Instituto de Investigacion Sanitaria (IDIS), Complejo Hospitalario Universitario de Santiago (CHUS/SERGAS). CIBER de Fisiopatologia de La Obesidad y Nutricion (CIBERobn), Instituto de Salud Carlos III, Madrid, Spain; 8 Gastroenterology and Gastrointestinal Endoscopy Unit, Vita-Salute San Raffaele University, IRCCS San Raffaele Scientific Institute, Milan, Italy; 9 Calcium Metabolism and Osteoporosis Program, American University of Beirut-Medical Center, Beirut, Lebanon; 10 Department of Human Sciences and Promotion of the Quality of Life, San Raffaele Roma Open University, Via Di Val Cannuta 247, 00166 Rome, Italy; 11 Experimental Medicine Department, Sapienza University, Rome, Italy; 12 Department of Medicine, School of Clinical Sciences, Monash University, Clayton, Australia; 13 Rheumatology Unit, University of Verona, Verona, Italy; 14 Department of Medicine, Université Laval, Quebec City, Canada; 15 Gastroenterology, Hepatology and Nutrition Division, Department of Life, Health and Environmental Sciences, University of L’Aquila, L’Aquila, Italy; 16 Department of Clinical and Experimental Medicine, University of Pisa, Pisa, Italy; 17 Kravis Center for Clinical Cardiovascular Health at Mount Sinai Heart, Icahn School of Medicine at Mount Sinai, New York, NY 10029 USA; 18 Sapienza Rome University, Rome, Italy; 19 Service of Bone Diseases, Geneva University Hospitals and Faculty of Medicine, Geneva, Switzerland; 20 Obesity and Lipodystrophy Center, University Hospital of Pisa, Pisa, Italy; 21 Department of Medicine, Medical College of Wisconsin, Milwaukee, WI USA; 22 Vitamin D Standardization Program, Havre de Grace, MD USA; 23 Institute of Public Health and Clinical Nutrition, University of Eastern Finland, Kuopio, Finland; 24 Department of Medicine and Surgery, Research Unit of Endocrinology and Diabetes, Università Campus Bio-Medico di Roma, Via Alvaro del Portillo, 21 - 00128 Roma, Italy; 25 Fondazione Policlinico Universitario Campus Bio-Medico, Via Alvaro del Portillo, 200 - 00128 Roma, Italy; 26 University of California, San Francisco and the San Francisco Veterans Affairs Health Care System, San Francisco, USA; 27 Department of Medicine, Endocrinology Division, Vagelos College of Physicians and Surgeons Columbia University, New York, NY USA

**Correction to: Reviews in Endocrine and Metabolic Disorders**

10.1007/s11154-023-09831-3

The article “Vitamin D status and supplementation before and after Bariatric Surgery: Recommendations based on a systematic review and meta‑analysis”, written by Andrea Giustina, Luigi di Filippo, Antonio Facciorusso, Robert A. Adler, Neil Binkley, Jens Bollerslev, Roger Bouillon, Felipe F. Casanueva, Giulia Martina Cavestro, Marlene Chakhtoura, Caterina Conte, Lorenzo M. Donini, Peter R. Ebeling, Angelo Fassio, Stefano Frara, Claudia Gagnon, Giovanni Latella, Claudio Marcocci, Jeffrey I. Mechanick, Salvatore Minisola, René Rizzoli, Ferruccio Santini, Joseph L. Shaker, Christopher Sempos, Fabio Massimo Ulivieri, Jyrki K. Virtanen, Nicola Napoli, Anne L. Schafer, John P. Bilezikian, was originally published electronically on the publisher’s internet portal on September 04, 2023 without open access. With the author(s)’ decision to opt for Open Choice the copyright of the article changed on September 09, 2023 to © The Author(s) 2023 and the article is forthwith distributed under a Creative Commons Attribution 4.0 International License, which permits use, sharing, adaptation, distribution and reproduction in any medium or format, as long as you give appropriate credit to the original author(s) and the source, provide a link to the Creative Commons licence, and indicate if changes were made. The images or other third party material in this article are included in the article’s Creative Commons licence, unless indicated otherwise in a credit line to the material. If material is not included in the article’s Creative Commons licence and your intended use is not permitted by statutory regulation or exceeds the permitted use, you will need to obtain permission directly from the copyright holder. To view a copy of this licence, visit http://creativecommons.org/licenses/by/4.0.

The original article has been corrected.

