# Supplementary material for: Vitamin D status and supplementation before and after Bariatric Surgery: Recommendations based on a systematic review and meta-analysis
Source: Rev Endocr Metab Disord. 2023 Sep 4;24(6):1011–29. doi: 10.1007/s11154-023-09831-3 (PMC10698146; doi:10.1007/s11154-023-09831-3)
Supplement: Supplementary file 1 — Supplementary file1 (DOCX 329 KB) [file 11154_2023_9831_MOESM1_ESM.docx]

**Supplementary materials**

*Keywords and standardized index terms used for the search strategy.*

"Vitamin D"[Mesh] OR “vitamin D”[tiab] OR “vitamin D level*”[tw] OR “Vitamin D serum level*”[tw] OR “Vitamin D absorption”[tw] OR “Vitamin D metabolism”[tw] OR “Vitamin D threshold”[tw] AND "Postoperative Complications"[Mesh] OR "Bariatric Surgery"[Mesh] OR "surgery" [Subheading] OR "Gastric Bypass"[Mesh] OR "Gastrectomy"[Mesh] OR “bariatric surgery success”[tw] OR “bariatric surgery”[tiab] OR “fat mass loss”[tw] OR “surgery”[tw] OR “post surgery”[tw] OR “post-surgery”[tw] OR “gastric bypass”[tw] OR “sleeve gastrectomy”[tw] AND "Weight Loss/physiology"[Mesh] OR ("Vitamin D Deficiency/blood"[Mesh] OR "Vitamin D Deficiency/physiopathology"[Mesh] OR "Vitamin D Deficiency/surgery"[Mesh]) OR ("Obesity, Morbid/blood"[Mesh] OR "Obesity, Morbid/physiopathology"[Mesh] OR "Obesity, Morbid/surgery"[Mesh]) OR "Calcium/therapeutic use"[Mesh] OR "Body Composition"[Mesh] OR "Vitamins/therapeutic use"[Mesh]OR "Obesity Management"[Mesh] OR "Obesity"[Mesh] OR "Parathyroid Hormone"[Mesh] OR "Body Mass Index"[Mesh] OR "Adipokines"[Mesh] OR "Gastrointestinal Microbiome"[Mesh] OR "Bone Diseases, Metabolic"[Mesh] OR "Osteoporosis"[Mesh] OR "Cholecalciferol"[Mesh] OR "Insulin Resistance"[Mesh] OR "Short Bowel Syndrome"[Mesh] OR "Adiposity"[Mesh] OR "Osteomalacia"[Mesh] OR "Malnutrition"[Mesh] OR "Diet, Fat-Restricted"[Mesh] OR "Dietary Supplements"[Mesh] OR "Hyperparathyroidism, Secondary"[Mesh] OR "cationic protein, urate-calcium oxalate stone, human" [Supplementary Concept] OR "Body Weight Changes"[Mesh] OR “calcium absorption”[tw] OR Obesity[tw] OR “Parathyroid hormone”[tw] OR BMI[tw] OR Adipokine*[tw] OR “Gut microbiota”[tw] OR Osteopenia[tw] OR Osteoporosis[tw] OR “Bone metabolism”[tw] OR “liver function*”[tw] OR Kidney*[tw] OR “Physical activity”[tw] OR “muscle mass loss”[tw] OR “Bone mass reduction”[tw] OR Cholecalciferol[tw] OR Calcifediol*[tw] OR “Energy balance”[tw] OR “Short bowel syndrome”[tw] OR “bile acid*”[tw] OR “Insulin resistance”[tw] “HOMA index”[tw] OR Fracture*[tw] OR DXA[tw] OR Adiposity[tw] OR Osteomalacia[tw] OR undernutrition[tw] OR malabsorption[tw] “Lean mass”[tw] OR Diet[tw] OR “Nutritional supplementation”[tw] OR “Secondary hyperparathyroidism”[tw]OR “Urinary calcium”[tw] OR “Body weight change”[tw] OR “bone marrow fat”[tw]”. Section C: "Vitamin D"[Mesh] OR "Ergocalciferols"[Mesh] OR "Cholecalciferol"[Mesh] OR "Calcifediol"[Mesh] OR “vitamin D”[tiab] OR calcitriol[tw] OR “25 hydroxyvitamin d 3”[tw] OR ergocalciferol[tw] OR cholecalciferol[tw] AND "Bariatric Surgery"[Mesh] OR "surgery" [Subheading] OR "Gastric Bypass"[Mesh] OR "Gastrectomy"[Mesh] OR "Jejunoileal Bypass"[Mesh] OR "Gastroplasty"[Mesh] OR “bariatric surgery”[tw] OR “sleeve gastrectomy”[tw] OR “gastric bypass”[tw] OR “jejunoileal bypass”[tw] OR “gastroplasty”[tw].

**Supplementary table 1.** Quality assessment of the observational and interventional studies included in the key clinical question #1 “Should 25(OH)D concentrations be assessed before bariatric surgery?”.

| Quality assessment of the studies included in the key clinical question #1 | | | | | | | | | | | |  |
| --- | --- | --- | --- | --- | --- | --- | --- | --- | --- | --- | --- | --- |
| Retrospective and prospective case–control studies^a^ | | | | | | | | | | | |  |
|  |  | | Selection | | | Comparability | | Outcome | | Overall quality | |  |
| Weiner 2020 (43) | | | *** | | | NA | | ** | | H | |  |
| Caron 2017 (44) | | | ** | | | NA | | ** | | M | |  |
| Signori 2010 (45) | | | ** | | | NA | | ** | | M | |  |
| Coupaye 2013 (46) | | | ** | | | NA | | ** | | M | |  |
| Sanchez 2005 (47) | | | ** | | | NA | | ** | | M | |  |
| Sinha 2011 (48) | | | ** | | | NA | | ** | | M | |  |
| Mahlay 2008 (49) | | | ** | | | NA | | ** | | M | |  |
| Fleischer 2008 (50) | | | ** | | | NA | | ** | | M | |  |
| Lanzarini 2015 (51) | | | ** | | | NA | | ** | | M | |  |
| Fish 2010 (53) | | | ** | | | NA | | ** | | M | |  |
| Wang 2016 (54) | | | ** | | | NA | | ** | | M | |  |
| Jin 2009 (55) | | | ** | | | NA | | ** | | M | |  |
| Gemmel 2009 (56) | | | ** | | | NA | | ** | | M | |  |
| Beckman 2013 (57) | | | ** | | | NA | | ** | | M | |  |
| Carlin 2006 (I) (58) | | | ** | | | NA | | ** | | M | |  |
| Carlin 2006 (II) (59) | | | ** | | | NA | | ** | | M | |  |
| Gehrer 2010 (60) | | | ** | | | NA | | ** | | M | |  |
| Randomized controlled trials^b^ | | | | | | | | | | | |  |
|  | | **1** | | **2** | **3** | | **4** | | **5** | | **6** |  |
| Sayadi 2019 (52) | | L | | H | H | | L | | U | | L |  |
| L, low; H, high; U, unclear; M, moderate.  ^a^ Study quality assessment performed by means of Newcastle/Ottawa scale (each asterisk represents if the respective items composing each criterion within the subsection was satisfied; every item satisfied is signed with one asterisk).  ^b^ Cochrane Collaboration’s tool for assessing the risk of bias across 7 domains: 1 (Random sequence generation), 2 (Allocation concealment), 3 (Blinding of participants and personnel), 4 (Blinding of outcome assessment), 5 (Incomplete outcome data), 6 (Selective reporting) and 7 (Other bias). | | | | | | | | | | | | |

**Supplementary table 2.** Quality assessment of the observational and interventional studies included in the key clinical question #2 “Should 25(OH)D concentrations be assessed after bariatric surgery? Do 25(OH)D concentrations change after bariatric surgery without specific postoperative supplementation?”.

| Quality assessment of the studies included in the key clinical question #2 | | | | | |  |
| --- | --- | --- | --- | --- | --- | --- |
|  |  | Selection | Comparability | Outcome | Overall quality |  |
| Johnson 2006 (61) | | ** | NA | * | L |  |
| Brolin 2002 (64) | | ** | NA | * | L |  |
| Newbury 2003 (63) | | ** | NA | * | L |  |
| de Campos 2008 (62) | | ** | NA | * | L |  |
| L, low; H, high; U, unclear; M, moderate; NA, not available.  ^a^ Study quality assessment performed by means of Newcastle/Ottawa scale (each asterisk represents if the respective items composing each criterion within the subsection was satisfied; every item satisfied is signed with one asterisk). | | | | | | |

**Supplementary table 3.** Quality assessment of the observational and interventional studies included in the key clinical question #3 “Is there a difference between restrictive and malabsorptive surgery in postoperative vitamin D status?”.

| Quality assessment of the studies included in the key clinical question #3 | | | | | | | | | | | |  |
| --- | --- | --- | --- | --- | --- | --- | --- | --- | --- | --- | --- | --- |
| Retrospective and prospective case–control studies^a^ | | | | | | | | | | | | |
|  |  | | Selection | | | Comparability | | Outcome | | Overall quality | |  |
| Fish 2010 (53) | | | *** | | | * | | * | | M | |  |
| Lanzarini 2015 (51) | | | *** | | | * | | * | | M | |  |
| Vilarrasa 2013 (66) | | | *** | | | * | | * | | M | |  |
| Gehrer 2010 (60) | | | ** | | | NA | | * | | L | |  |
| Randomized controlled trials^b^ | | | | | | | | | | | | |
|  | | **1** | | **2** | **3** | | **4** | | **5** | | **6** | |
| Vix 2014 (65) | | L | | H | H | | L | | L | | L | |
| L, low; H, high; U, unclear; M, moderate; NA, not available.  ^a^ Study quality assessment performed by means of Newcastle/Ottawa scale (each asterisk represents if the respective items composing each criterion within the subsection was satisfied; every item satisfied is signed with one asterisk).  ^b^ Cochrane Collaboration’s tool for assessing the risk of bias across 7 domains: 1 (Random sequence generation), 2 (Allocation concealment), 3 (Blinding of participants and personnel), 4 (Blinding of outcome assessment), 5 (Incomplete outcome data), 6 (Selective reporting) and 7 (Other bias). | | | | | | | | | | | | |

**Supplementary table 4**. Quality assessment of the observational and interventional studies included in the key clinical questions #1 “What dose of vitamin D3 is necessary for most people who have undergone bariatric surgery (RYGB, SG, AGB, BPD-DS) to achieve and maintain 25(OH)D levels of ≥30 ng/mL?” and #2 “Does the type of bariatric surgery influence the dose of vitamin D supplement required?” of the second topic.

| Retrospective and prospective case–control studies^a^ | | | | | | | | | |
| --- | --- | --- | --- | --- | --- | --- | --- | --- | --- |
|  | Selection | | | Comparability | | Outcome | | Overall quality | |
| Aasheim 2012 (67) | ** | | | NA | | *** | | M | |
| Bagioni 2014 (68) | ** | | | NA | | *** | | M | |
| Bagioni 2017 (69) | ** | | | NA | | *** | | M | |
| Bandstein 2015 (70) | ** | | | NA | | *** | | M | |
| Bourbour 2021 (71) | ** | | | NA | | *** | | M | |
| Boyce 2015 (72) | ** | | | NA | | ** | | M | |
| Carlin 2009 (73) | *** | | | NA | | *** | | M | |
| Caron 2017 (44) | ** | | | NA | | *** | | M | |
| Chan 2015 (74) | ** | | | NA | | *** | | M | |
| Costa 2015 (75) | ** | | | NA | | *** | | M | |
| Coupaye 2014 (76) | ** | | | NA | | ** | | M | |
| Da rosa 2013 (77) | ** | | | NA | | *** | | M | |
| Dos santos 2021 (79) | ** | | | NA | | ** | | M | |
| Einarsdóttir 2010 (80) | ** | | | NA | | *** | | M | |
| Elhag 2022 (81) | ** | | | NA | | *** | | M | |
| Flores 2010 (82) | ** | | | NA | | *** | | M | |
| Homan 2018 (86) | ** | | | NA | | *** | | M | |
| James 2016 (89) | ** | | | NA | | *** | | M | |
| Lanzarini 2015 (51) | ** | | | ** | | *** | | H | |
| Nelson 2007 (91) | *** | | | ** | | *** | | H | |
| Smelt 2019 (94) | ** | | | NA | | *** | | M | |
| Strain 2017 (95) | ** | | | NA | | ** | | M | |
| Syn 2020 (95) | ** | | | NA | | *** | | M | |
| Topart 2014 (97) | ** | | | NA | | *** | | M | |
| Tsiftsis 2009 (98) | ** | | | NA | | *** | | M | |
| Volonakis 2020 (99) | ** | | | NA | | *** | | M | |
| Weiner 2020 (100) | ** | | | NA | | ** | | M | |
| Yu 2015 (102) | ** | | | NA | | *** | | M | |
| Randomized controlled trials^b^ | | | | | | | | | |
|  | **1** | **2** | **3** | | **4** | | **5** | | **6** |
| Vix 2014 (65) | L | H | H | | **L** | | L | | L |
| Dogan 2014 (78) | L | L | L | | L | | L | | L |
| Galyean 2022 (83) | L | U | U | | L | | L | | L |
| Goldner 2008 (84) | L | U | L | | H | | L | | L |
| Heusschen 2020 (85) | L | L | U | | H | | H | | L |
| Hultin 2018 (87) | L | U | U | | L | | L | | L |
| Ikramuddin 2015 (88) | L | L | L | | L | | L | | L |
| Luger 2018 (90) | L | U | U | | H | | L | | L |
| Perin 2018 (92) | L | H | H | | H | | L | | L |
| Ruiz-tovar 2016 (93) | L | U | U | | L | | L | | L |
| Wolf 2016 (101) | L | L | L | | L | | L | | L |
| L, Low; H, High; U, Unclear; M, Moderate; NA, not available.  ^a^ Study quality assessment performed by means of Newcastle/Ottawa scale (each asterisk represents if the respective items composing each criterion within the subsection was satisfied; every item satisfied is signed with one asterisk).  ^b^ Cochrane collaboration’s tool for assessing the risk of bias across 7 domains: 1 (random sequence generation), 2 (allocation concealment), 3 (blinding of participants and personnel), 4 (blinding of outcome assessment), 5 (incomplete outcome data), 6 (selective reporting) and 7 (other bias). | | | | | | | | | |

**Supplementary Figure 1**. Pooled analysis of preoperative 25(OH)D levels (ng/mL) in patients who underwent bariatric surgery.





**Supplementary Figure 2.** Visual assessment of the funnel plot for publication bias of clinical question #1.





**Supplementary Figure 3.** Pooled analysis of postoperative of 25(OH)D <30 ng/mL (A) and <20 ng/mL (B) in patients after bariatric surgery without postoperative vitamin D routine supplementation.





**Supplementary Figure 4.** Visual assessment of the funnel plot for publication bias of clinical question #2.





**Supplementary Figure 5.** Visual assessment of the funnel plot for publication bias of clinical question #3.





**Supplementary Figure 6.** Visual assessment of the funnel plot for publication bias of clinical questions #1 and #2 of the second topic.
